# Location and extent of cavernous transformation of the portal vein dictates different visceral side revascularization in Meso-Rex bypass

**DOI:** 10.1186/s12893-023-02168-3

**Published:** 2023-09-13

**Authors:** Rui Tang, Guangdong Wu, Qiang Yu, Xuan Tong, Xiangfei Meng, Yucheng Hou, Xin Huang, Abudusalamu Aini, Lihan Yu, Weidong Duan, Qian Lu, Jun Yan

**Affiliations:** 1 Hepatopancreatobiliary Center, Beijing Tsinghua Changgung Hospital, Key Laboratory of Digital Intelligence Hepatology (Chinese Ministry of Education), School of Clinical Medicine, Institute for Precision Medicine, Tsinghua University, No. 168 Litang Road, Beijing, 102218 China; 2 Department of Hepatobiliary and Pancreatic Surgery, Chinese PLA General Hospital, No. 28 Fuxing Road, Beijing, 100853 China; 3 General Surgery Department, Lhasa People’s Hospital, Tibet Autonomous Region, Lhasa, China

**Keywords:** Portal vein cavernous transformation, Meso-rex bypass, Portal vein reconstruction, Vascular anastomosis, Portal vein thrombosis

## Abstract

**Background:**

As an emerging standard of care for portal vein cavernous transformation (PVCT), Meso-Rex bypass (MRB) has been complicated and variated. The study aim was to propose a new classification of PVCT to guide MRB operations.

**Methods:**

Demographic data, the extent of extrahepatic PVCT, surgical methods for visceral side revascularization, intraoperative blood loss, operating time, changes in visceral venous pressure before and after MRB, postoperative complications and the condition of bypass vessels after MRB were extracted retrospectively from the medical records of 19 patients.

**Results:**

The median age of the patients (13 males and 6 females) was 32.5 years, while two patients were underage. Causes of PVCT can be summarized as follows: thrombophilia such as dysfunction of antithrombin III or proteins C; secondary to abdominal surgeries; secondary to abdominal infection or traumatic intestinal obstruction, and unknown causes. Intraoperatively, the median operation time was 9.5 h (7–13 h), and the intraoperative blood loss was 300 mL (100-1,600 mL). Ten cases used autologous blood vessels while 10 used allogeneic blood vessels. The vascular anastomosis was divided into the following types according to the site and approach: Type (T) 1-PV pedicel type, T2-confluence type, T3-major visceral vascular type; and T4-collateral visceral vascular type. Furthermore, the visceral venous pressure before and after MRB dropped significantly from 36 cmH_2_O (28–44) to 24.5 cmH_2_O (15–31) (*P* < 0.01). Postoperatively, one patient had delayed wound healing, two developed biochemical pancreatic fistulae, one experienced lymphatic leakage, the former caused by heat damage of the pancreatic tissues, the latter by cutting lymphatic vessels in the mesentery or removing the local lymph nodes during the process of separating the superior mesenteric vein, and one was re-operated on for an intervening intestinal fistulae. Postoperative enhanced CT scans revealed a significant improvement in abdominal varix in the patients with patent bypass, and at the 1-year postoperative follow-up, enhanced CT scans of six patients showed that the long axis of the spleen was reduced by ≥ 2 cm.

**Conclusions:**

MRB can effectively reduce visceral venous pressure in patients with PVCT. It is feasible to determine the PVCT type according to the extent of involvement and to choose individualized visceral side revascularization performances.

## Background

As a rare pathological entity [1, 2], portal vein cavernous transformation (PVCT) occurs with long-standing portal vein thrombosis (PVT), which causes portal hypertension and occlusion of the portal vein (PV) leading to the development of sponge-like venous collaterals in and around the re-canalizing main PV, and an enlarged spleen on CT angiography [3]. Clinically, PVCT presents with recurrent gastroesophageal variceal bleeding and hematologic abnormalities, and the goal for management of PVCT is to effectively decompress venous congestion and hypertension along with the mesenteric and splenic components of the portal system and to safeguard adequate portal inflow to the liver [4].

At present, surgical treatment has been considered as the standard of care for PVCT; however, the operational procedure is relatively difficult because the vessels follow bizarre and non-anatomical courses and are susceptible to bleeding [5]. Compared to treatments including portosystemic shunt [6–8], paraesophagogastric devascularization, variceal banding ligation, splenorenal shunt [9], and sclerotherapy, the Rex-bypass shunt has emerged as a novel but effective surgical intervention for PVCT without additional liver lesions [10]. This procedure has currently been highlighted as being representatively similar to strategies for PVT management in liver transplantation [11].

PV reconstruction techniques with Meso-Rex bypass (MRB) creates a bypass between the superior mesenteric vein (SMV) and the left portal system, when the splanchnic venous blood circulation should be restored [12]. However, the methods for establishing MRB vary depending on the location and extent of cavernous transformation. In the present study, we report the different choices that were made based on the involved portion of extrahepatic PVCT for visceral side revascularization with either jumping or interposed vein graft for creating MRB and propose a new classification of PVCT to guide the operation of MRB.

## Methods

### Patients

Patients who underwent MRB from January 2013 to December 2020 were included retrospectively. To accommodate possible differences in understanding the procedure, MRB was defined as the establishment of a bypass either from PV pedicel, splenic vein (SV) or SMV, coronary vein, inferior mesenteric vein (IMV), or any other visceral venous vessel routed to the left PV with auto- or allograft vein. The extrahepatic portion and extent of PVCT were evaluated by preoperative enhanced CT/MRI and angiography. All methods in this study were carried out in accordance with relevant clinical guidelines and regulations. The study was based on the Declaration of Helsinki and was approved by the Ethics Committee of Beijing Tsinghua Changgung Hospital. Written informed consent of the enrolled patients or their legal guardians was obtained.

Inclusion criteria for this study were: [1] PVCT diagnosed by enhanced CT or MRI imaging; [2] angiography revealed normal left and right branches of intrahepatic PV and their confluence; [3] MRB was successfully performed; and [4] clinical data were complete and thorough. Exclusion criteria were: [1] patients who also presented with malignancies as one of the comorbidities; [2] simultaneous liver resection was also performed but was not intended to expose Rex recesses (liver tumor resection etc.); [3] liver cirrhosis suggested by imaging or pathology; or [4] PVCT involved either the left or right branches of the intrahepatic PV or their confluence.

The information on demographic data, the extent of extrahepatic PVCT, surgical methods for visceral side revascularization, intraoperative blood loss, operating times, changes in visceral venous pressure before and after MRB, postoperative complications and the condition of bypass vessels after MRB were extracted retrospectively from medical record reviews. The endpoint for postoperative follow-ups was June 2022.

### Statistical analysis

Data were analyzed using SPSS (ver. 23.0). Quantitative data with skewed distributions are expressed as medians and the range. Categorical data are given as absolute numbers. A *P*-value < 0.05 was considered to be statistically significant.

## Results

### General information

The present study involved 19 patients, including 13 males and 6 females with a median age of 32.7 years (range 6 to 68 years) including 2 underage patients. A total of 20 MRBs were performed because 1 patient underwent a MRB procedure twice. Causes of PVCT were: [1] 4 cases of thrombophilia such as dysfunction of antithrombin III or proteins C; [2] 4 cases secondary to abdominal surgeries, which were further stratified as 2 orthotopic liver transplantation, 1 ex vivo liver resection, 1 bile duct jejunum Roux-en-Y anastomosis; [3] 4 cases secondary to abdominal infection or traumatic intestinal obstruction, including 1 pancreatitis, 1 portal phlebitis secondary to appendicitis, 1 intestinal obstruction, 1 liver trauma; and [4] 8 cases with unknown causes.

Intraoperatively, the median operation time was 9.5 h (range 7 to 13 h) and the intraoperative blood loss was 300 mL (100-1,600 mL). Ten cases used autologous blood vessels and 10 used allogeneic blood vessels. The autologous blood vessels were harvested from the right internal jugular vein or coronary vein. The allogeneic vessels began to be used in our hospital from March 2016, and for MRBs the iliac vein was harvested from donation after cardiac or brain death and implanted within 3 days.

### Surgical performances for visceral side revascularization

**Fig. 1:**
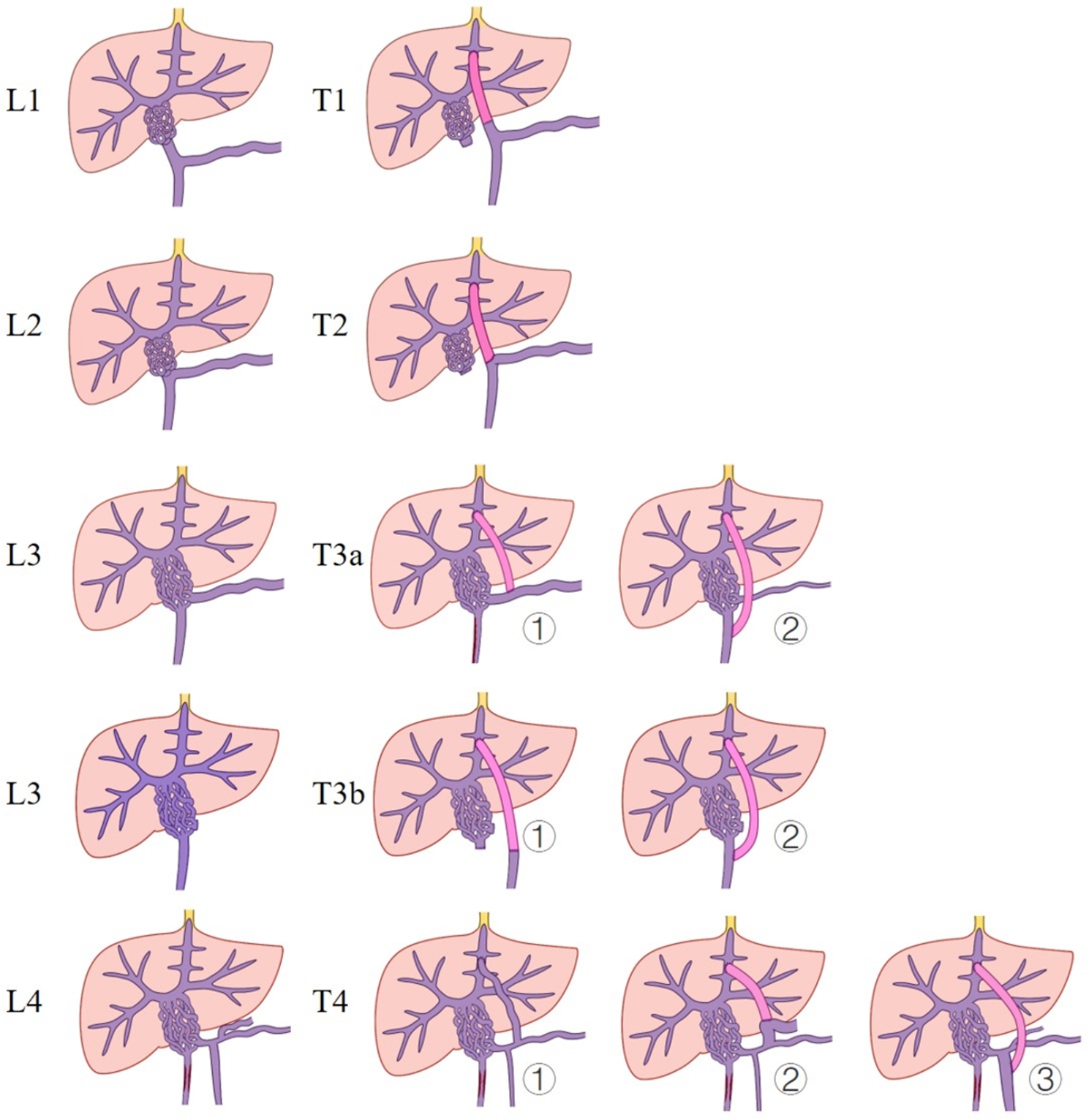
Classification of levels and types for PVCT according to the sites and approaches of vascular anastomosis. L, level; PVCT, portal vein cavernous transformation; T, type

**Fig. 2:**
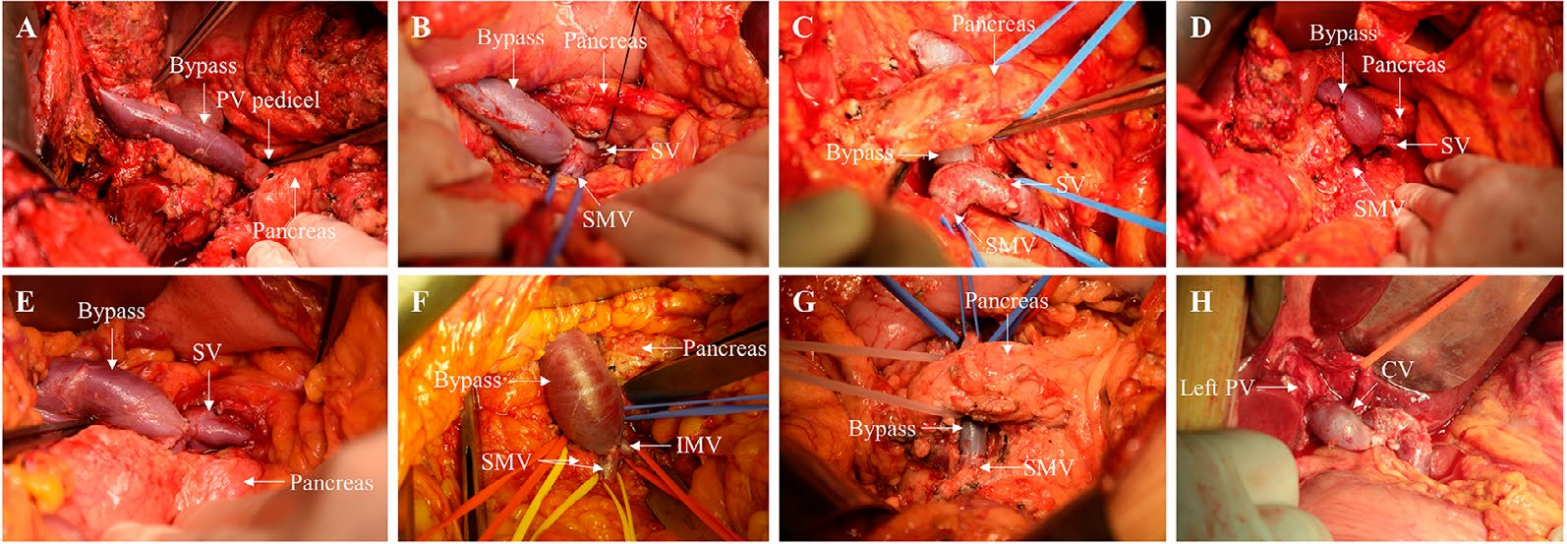
Different vascular anastomosis methods and vascular bridging paths. Different surgical procedures were designed according to the extent of portal vein spongiosis and the anatomical condition of the dorsal pancreas. (A), T1R3; (B), T2R1; (C), T2R2; (D), T3a①R1; (E), T3a①R3; (F), T3a②R1; (G), T3b①R2; (H) T4①. T, type; R, route

The vascular anastomosis was divided into the following types according to the site and approach. Type (T) 1 as PV pedicel type was performed on 4 cases with an end-to-end anastomosis being made to the PV root that was also the bridge that extended to the confluence of the SMV and SV. T2 as a confluence type was performed in 4 cases, which had end-to-end anastomosis made directly to the confluence of the SMV and SV, where the PV trunk was absent. T3 as a major visceral vascular type could further be divided into T3a and T3b; T3a was performed in 9 cases as end-to-side anastomosis being made to the SMV or SV trunk, which could also be subdivided as [1] T3a, SV dominant type for 5 cases, and [2] T3a, SMV dominant type for 4 cases. Furthermore, 1 case was T3b, which included either end-to-end or end-to-side anastomosis being made to the SMV trunk after splenectomy (T3b). T4, a collateral visceral vascular type: 2 cases, anastomosis was made to the coronary vein or IMV (T4) (Figs. 1 and 2).

**Fig. 3:**
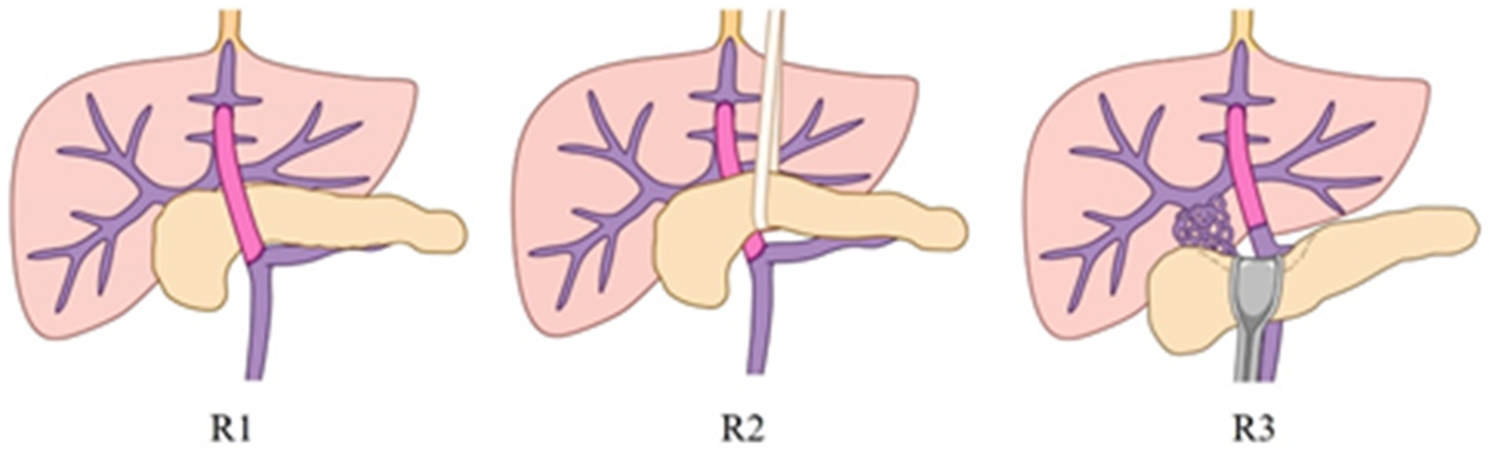
Positional relationship between bypass vessel routes and the pancreas. Different vascular bridging routs. R1 is the retrogastric tunnel route in the ventral side of the pancreas. R2 is the pullout route on the dorsal side of the pancreas, and the bridging vessels completely passed through the dorsal side of the pancreas. R3 is the low dissection route, in which the bridging vessels did not need to pass through the dorsal side of the pancreas. R, route

Moreover, for T2 and T3, the surgical approach was selected based on dorsal pancreas conditions such as adhesion, the existence of varicose veins and deviation of the spleen vein to the superior or inferior margin of the pancreas. Intraoperatively, SMV and SV confluence, and the SMV or SV trunk could be isolated either from the inferior margin of the pancreas to the superior margin or directly from the superior margin. Therefore, according to the positional relationship between the bypass vessel and the pancreas, the surgical approach could be divided into the following three routes: retrogastric tunnel route as R1 was performed in 8 cases, which was identical to traditional MRB and had the reconstructed vessel bypass the ventral side of the pancreas; pullout route as R2 was performed in 5 cases, in which the dorsal pancreatic tunnel was exposed for vascular dissociation and anastomosis and the reconstructed vessel passed through the dorsal pancreas; R3 (low dissection route) was carried out in 5 cases, during which the PV trunk, SMV and SV confluence or SV trunk were isolated from the superior margin of the pancreas. Then all vessels anastomosed to the left PV directly, and the blood flow passed through the dorsal side of the pancreas (Fig. 3).

### Outcomes and complications

After the initial exploration of the abdominal cavity was completed, and after the bypass opened, the visceral venous pressure was measured twice via a central venous catheter which was inserted into the selected right gastroepiploic vein or branches of the SMV (such as the branch of the ileocolic vein).

**Fig. 4:**
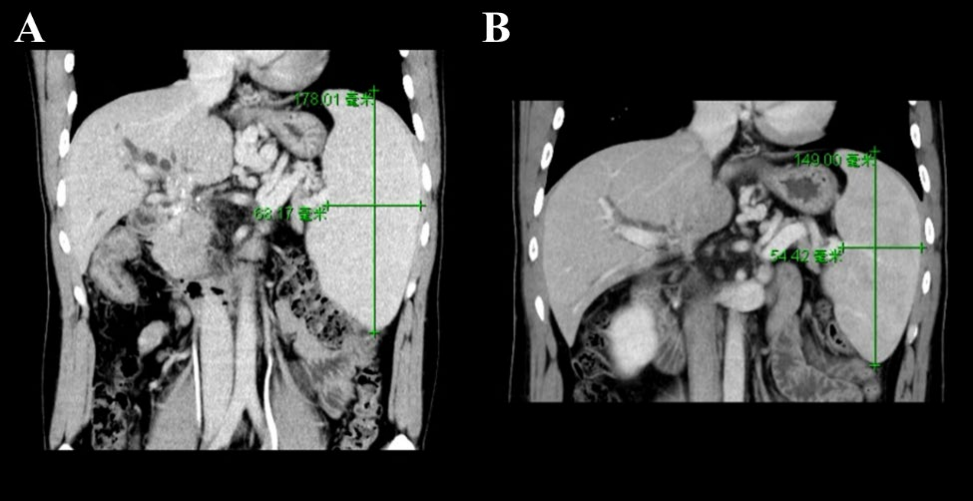
At the 1-year postoperative follow-up, enhanced CT scans of 6 patients showed that the long axis of the spleen was reduced to ≥ 2 cm. The comparison of splenic volume between a preoperative (A) and postoperative (B) MESO-REX patient showed that the splenic volume decreased significantly after the operation suggesting that the MESO-REX intervention was effective

In this case series, the visceral venous pressure before and after MRB dropped significantly from 36 cmH_2_O (28–44) to 24.5 cmH_2_O (15–31) (*P* < 0.01). Postoperative enhanced CT scans revealed a significant improvement in abdominal varix in patients with patent bypass, and at the 1-year postoperative follow-up, enhanced CT scans of 6 patients showed that the long axis of the spleen was reduced to ≥ 2 cm (Fig. 4).

Four cases had postoperative Clavien-Dindo grade 1–2 with 1 delayed wound healing, 2 biochemical pancreatic fistulae and 1 lymphatic leakage, while 2 cases had non-vascular related Clavien-Dindo grade 3–4 as a result of intestinal fistula or bile leakage accompanied by abdominal infection, which had previously been managed by surgical intervention. The biochemical pancreatic fistulae may have been caused by the heat damage to the pancreatic tissues when the SMVs were isolated from the inferior margins or the dorsal side of the pancreas, but the 2 patients were both cured without any treatment. Lymphatic leakage may have been caused by cutting off the lymphatic vessels in the mesentery or removal of the local lymph nodes during the process of separating the SMV. The patient recovered quickly by fasting and soon resumed a normal diet.

A total of 11 MRBs had patent bypass vessels at the last follow-ups, 2 of which had stenosis but were corrected by stenting. However, 9 MRBs suffered embolization along the bypass vessels, 5 of which received embolectomy and underwent revascularization or interventional therapy with 1 case having a second MRB after interventional therapy and 1 patient failed to open the blood vessel through interventional therapy, while 4 patients did not undergo any invasive intervention for their re-occlusion. The bypass vessel occlusion occurred at a median time of 6 months (0–40 months) after the operation, and the vascular patency duration was 34.5 months (0-101 months). A total of 7 cases had occlusion due to stenosis, when the diameter of the anastomosis was < 4 mm as revealed by postoperative enhanced CT scans. On the other hand, both patients who received coronary veins had bypass embolization, and the occlusion time was 36 months after the operation. Compared to autologous blood vessels, allogeneic blood vessels had a patency rate of 60% vs. 50% and a patency duration of 36.5 months (3–81) vs. 53 months (0-101)], but the differences were not significant (*P* = 0.1).

Among the 9 patients with vascular occlusion, 5 were L1, 2 were L2, and one each for L3 and L4. According to the classification of surgical methods, there were 4 occlusive cases (100%) in T1 with a median patent time of 22.5 (15–32) months, 2 cases each in T2 and T4, and 1 case in T3a. In R3, 4 cases (80%) were occluded with a median patent time of 26 (3–85) months. Bypass occlusion occurred in 10% (n = 1/10) among T3 operations with a median patent time of 60.5 (3-101) months (longer than T1 with a statistically significance, *P* = 0.037), and 12.5% (n = 1/8) through R1 path with a median patent time of 46 (15–81) months (longer than R3 but without statistical significance, *P* = 0.150).

## Discussion

PVCT is a collateral circulation to the hepatic vein formed after extrahepatic PV obstruction [13]. For managing this disease process, the Meso-Rex shunt, which was first described by de Ville de Goyet as interposing a mesenteric-left portal shunt at the level of the umbilical portion of the left PV system (Rex’s recessus) as a solution to the cause of portal hypertension, emerged to be an effective treatment to significantly reduce the portal pressure and the degree of esophagogastric varices and improve hypersplenism [11, 12].

Surgically, Meso-Rex shunting could be complicated and variated by the location and extent of PVCT, so a new approach was attempted in our practice to tackle the problem [14, 15]. Based upon our practice, PVCT might be further classified into different levels corresponding to the different sites of lateral visceral revascularization (Fig. 1). Level 1 (L`Z) was designated as PVCT that involved the PV trunk with a short PV pedicle remnant, which was similar to Yerdel T2 PVT; and in operation the PV stump was isolated from the superior margin of the pancreas using a low “dissection technique”, then visceral side revascularization (T1R3, or T2R1-3, or T3R1-2) was performed to permit SMV and SV inflow through PV stump-bypass-Rex to enter the liver. L2 corresponded to PVCT involved the PV trunk with little PV pedicle, which was similar to Yerdel T3 PVT, and operationally, the junction of the SMV and SV was just free of PVCT with the employment of the “pullout technique” at the inferior margin of the pancreas, or the adoption of the “low dissection” route at the superior margin of the pancreas for performing visceral side revascularization at the confluence of the SMV and SV. However, if the dorsal channel of the pancreas could not be completely exposed, then the bypass needed to pass through the ventral side of the pancreas (T2R1-3). Moreover, T3R1-3 could also be used as an alternative. L3 was assigned to PVCT involving the PV trunk and SMV/SV confluence, or if only the SMV trunk remained after splenectomy, which included the cases T3R1-3. Lastly, L4 was due to unavailability of the SMV or SV trunk as a result of either PVCT or thrombus formation, but with enlarged splanchnic collateral veins such as the coronary vein or IMV, which could be selected for anastomosis when blood flow was sufficient (T4, R1 was chosen when using IMV). The surgical performance after revascularization is shown in Fig. 3. In summary, the principle of revascularization is to ensure fluent visceral blood outflow to achieve the purpose of decompressing the portal system and ensuring adequate inflow (Table 1; Figs. 1 and 2) [16, 17].

**Table 1:** Yerdel Classification (2000)

Grade	Characteristic
**Grade 1**	Minimally or partially thrombosed PV where thrombus is mild or confined to < 50% of the lumen with or without extension into the SMV.
**Grade 2**	> 50% occlusion including total occlusion with or without minimal extension into the SMV.
**Grade 3**	Complete thrombosis of both the PV and proximal SMV.
**Grade 4**	Complete thrombosis of the PV and both proximal and distal SMV.

Abbreviations: PV: portal vein; SMV: superior mesenteric vein

MRBs, on the one hand, can be performed with interposed vein grafting that is described as establishing an anastomosis between a bypass vessel and splanchnic vein with the closure of the original blood flow. On the other hand, it could be created by jumping vein grafting i.e. to fashion an anastomosis between the bypass vessel and splanchnic vein without disturbing the original blood flow [18–20]. We recommend the interposed vein graft approach (T1-2), which was end-to-end anastomosis of the PV stump or the confluence of the SMV and SV to the left PV to achieve sufficient visceral circulation. Low dissection or pullout routes were usually applied in liver transplants for PVT, which direct visceral blood to the liver through the bypass, having the advantage of preserving the anatomical circulation. However, the operation was relatively complex, especially when the patient had a history of abdominal infection or chronic pancreatitis. The low dissection route required dissection near the superior margin of the pancreas close to the PVCT lesion, which was vulnerable to massive bleeding. The pullout route, on the other hand, required dissection of the confluence of the SMV and SV behind the pancreas. The bypass vessel in the conventional pullout route was located behind the pancreas (R2) but was most difficult to be dissected in PVCT [21]. But in some patients, the retrogastric route (R1) could be used instead of dissecting the retropancreatic tunnel. A careful dissection of the superior and inferior pancreaticoduodenal veins was always required for both the low dissection and pullout routes [22]. To guarantee satisfactory hepatopetal flow, suitable vascular diameter, sufficient blood flow and no compression or distortion of the vessel are essential techniques to prevent patients developing postoperative thrombus or occlusion [23–25]. The risk factors of thrombophilia, hypercoagulability or potential liver disease should be carefully explored for better surgical outcomes.

When only the SMV or SV trunk could be used for jumping vein graft anastomosis, it is critical to thoroughly evaluate the adequacy of existing communicative branches of the SMV and SV for reducing the venous pressure of the stomach and spleen. In addition, the dominant draining vessel should be identified before anastomosis to ensure sufficient bypass flow [26]. The end-to-side anastomosis was a commonly performed procedure but was characterized as the bypass being angled with the PV to cause the direction of blood inflow not being straight into the PV [27, 28]. Also, using coronary vein or IMV for anastomosis can result in insufficient blood flow because they are not major visceral vessels, and inherently lead to long-term inadequacy in blood inflow to the liver and persistent low in portal pressure. Thus, we recommend to use this option only in the situation where the SMV or SV trunk are not available. Sufficient front flow could be accessed by de-clamping the grafted vein to evidence profuse blood flow and adequate pressure. The splenorenal shunt should be ligated to avoid portal steal. Preoperatively, enhanced CT and angiography can help to define the classification of PVCT, splanchnic blood flow and possible alternative sites of vascular anastomosis. A 3D reconstruction based on enhanced CT is helpful to understand the spatial structure of the abdominal vessels and to estimate the length of bypass vessels.

As a major cause of postoperative bypass occlusion, anastomotic or luminal stenosis might result due to contraction of the anastomosis or compression from inflammation and edema of the surrounding tissues. Decelerated blood flow and hypercoagulability also contribute to the onset of thrombosis in bypass vessels. Our study suggested that diameters of post-anastomotic vascular lumen > 4 mm would effectively reduce the risk of bypass vessel occlusion. Some studies have reported that the autologous internal jugular vein might maintain a better postoperative patency than freshly harvested allogeneic blood vessels, whereas the cryopreserved allogeneic blood vessels were the worst for achieving long-term potency in bypassed vessels. Similarly, the result from the present study show that the patency of allogeneic blood vessels was shorter than that of autologous blood vessels, which should be further verified due to late introduction of allogeneic blood vessels into our practice (after March 2016). In fact, the rates of vascular occlusion for these two different grafts were similar, as after excluding the cases with coronary vein for the bypassed vessel, the patency rates for both vessels were 60% (allogeneic) and 62.5% (autologous), respectively. Therefore, occlusion is still the major risk factor for failure of MRBs [29]. The increased prevalence of occlusions in T1 and R3 may be caused by the close proximity to unhealthy porta hepatis or diseased vessels. This implies that the ideal anastomosis location should be chosen for the procedure away from the regions close to the hepatoduodenal ligament or surrounding the pancreas, which are prone to postoperative inflammation. Although our results showed that T3 and R1 achieved relatively good patency rates, they were not sufficient to negate other surgical methods, perhaps due to the relatively small cohort size and many other cofactors.

Doppler ultrasonography should be performed immediately after reperfusion, and the blood flow in the bypassed vessels should be measured daily within the first week after the MRB operations. Most studies have suggested that continuing anticoagulation regimens should be maintained for at least three months, but this is still a controversial conjecture. Activated partial thromboplastin time should be maintained around 50–70 s when using heparin at the beginning, and then switch to a preventive dose of low-molecular-weight heparin or warfarin, and INR should be monitored and range between 1.5 and 2.5. The use of aspirin was also reported [30]. From our experience, we recommend consistent anticoagulant therapy with oral administration of warfarin or rivaroxaban for at least 6 months. For patients with coagulation dysfunction, such as thrombophilia (dysfunction of antithrombin III or proteins C) or patients who received stent implantation, life-long anticoagulation is always recommended. Three of our four patients with thrombophilia developed bypass vessel thrombosis, probably because none of them adhered to a regimen of long-time anticoagulant therapy.

## Conclusions

MRB can effectively reduce visceral venous pressure in patients with PVCT. It is feasible to determine PVCT types according to the extent of involvement and to choose individualized visceral side revascularization performances. Further studies should be conducted to investigate the patency rate and duration of this individualized bypass treatment comparing to present conventional approaches.

## Data Availability

The data that support the findings of this study are available from the corresponding author upon reasonable request.

## Abbreviations

IMV: inferior mesenteric vein
MRB: Meso-Rex bypass
PV: portal vein
PVCT: portal vein cavernous transformation
PVT: portal vein thrombosis
SMV: superior mesenteric vein
SV: splenic vein
T: type
